# Health-related quality of life in Chinese population with non-alcoholic fatty liver disease: a national multicenter survey

**DOI:** 10.1186/s12955-021-01778-w

**Published:** 2021-05-07

**Authors:** Rui Huang, Jian-Gao Fan, Jun-Ping Shi, Yi-Min Mao, Bing-Yuan Wang, Jing-Min Zhao, Lun-Gen Lu, Bi-Hui Zhong, Zheng-Sheng Zou, You-Qing Xu, Yi-Nong Ye, Long-Gen Liu, Lian-Jie Lin, Jian-Ping Li, Hui-Ying Rao, Lai Wei

**Affiliations:** 1 Peking University People’s Hospital, Peking University Hepatology Institute, Beijing Key Laboratory of Hepatitis C and Immunotherapy for Liver Diseases, Beijing International Cooperation Base for Science and Technology On NAFLD Diagnosis, No.11 Xizhimen South Street, Beijing, 100044 China; 2 Department of Digestive, Xinhua Hospital Affiliated to Shanghai Jiao Tong University School of Medicine, Shanghai, China; 3 Department of Hepatology, The Affiliated Hospital of Hangzhou Normal University, Hangzhou, China; 4 Department of Digestive, Renji Hospital Affiliated to Shanghai Jiao Tong University School of Medicine, Shanghai, China; 5 Department of Digestive, The First Hospital of China Medical University, Shenyang, China; 6 Department of Pathology, The Fifth Medical Center of PLA General Hospital, Beijing, China; 7 Department of Digestive, The First People’s Hospital Affiliated to Shanghai Jiao Tong University School of Medicine, Shanghai, China; 8 Department of DigestiveThe First Affiliated Hospital, Sun Yat-Sen University, Guangzhou, China; 9 Department of DigestiveBeijing Tiantan Hospital, Capital Medical University, Beijing, China; 10 Department of Infectious Disease, The First Hospital of Fushan, Fushan, China; 11 Department of Hepatology, The Third People’s Hospital, Changzhou, China; 12 Department of Digestive, Shengjing Hospital of China Medical University, Shenyang, China; 13 China Resources Sanjiu Medical and Pharmaceutical Co., Ltd, Shenzhen, China; 14 Hepatopancreatobiliary Center, Beijing Tsinghua Changgung Hospital, Tsinghua University, No.168, Litang Road, Changping District, Beijing, 102218 China

**Keywords:** Chronic liver disease questionnaire, Health-related quality of life, Non-alcoholic fatty liver disease, Risk factors

## Abstract

**Background:**

Health Related Quality of Life (HRQL) is a multi-dimensional construct that can comprehensively evaluate the patient’s health status, including physical, emotional, mental and social well-being. In this study, we aimed to evaluate the impact of non-alcoholic fatty liver disease (NAFLD) on HRQL in a Chinese population.

**Methods:**

In this national multicenter cross-sectional survey, patients with NAFLD were enrolled. Chronic Liver Disease Questionnaire (CLDQ)-NAFLD was used to qualify HRQL. Univariate and multivariate analysis were used to identify independent risk factors of HRQL.

**Results:**

A total of 5181 patients with NAFLD from 90 centers were enrolled in this study (mean age, 43.8 ± 13.3 years; male, 65.8%). The overall CLDQ score was 5.66 ± 0.89. Multivariate logistic regression analysis showed that body mass index (BMI: HR, 1.642; 95% CI, 1.330–2.026), alanine transaminase (ALT: HR, 1.006; 95% CI, 1.001–1.011), triglyceride (HR, 1.184; 95% CI, 1.074–1.305), disease severity (HR, 3.203; 95% CI, 1.418–7.232) and cardiovascular disease (HR, 4.305; 95% CI, 2.074–8.939) were independent risk factors for overall CLDQ score. In the logistic analyses of individual domain, BMI and triglyceride were independent risk factors of all domains. ALT, disease severity, diabetes, depression and cardiovascular disease were influencing factors for the CLDQ score of several domains.

**Conclusions:**

This national multicenter cross-sectional survey in China indicated that the HRQL in patients with NAFLD was impaired. HRQL was found to be significantly associated with sociodemographic and clinical factors. Attention should be paid to the optimally managing care of patients with NAFLD to improve their HRQL.

## Background

Non-alcoholic fatty liver disease (NAFLD) is common chronic liver disease, which is characterized by the abnormal fat accumulation in liver without excessive alcohol intake [1]. This disease usually starts with simple steatosis, and then progress to non-alcoholic steatohepatitis (NASH) accompanied by different degrees of inflammation, with or without fibrosis, and ultimately develops to cirrhosis and hepatocellular carcinoma [2]. The global prevalence of NAFLD based on imaging is around 25% [3, 4]. With the increasing prevalence of obesity and diabetes in China, it is estimated that the national prevalence of NAFLD is 29.2%, and the number of patients with NAFLD in China may account for half of patients with NAFLD worldwide [5].

Health Related Quality of Life (HRQL) is a multi-dimensional construct that can comprehensively evaluate the patient’s health status, including physical, emotional, mental and social well-being [6]. The Chronic Liver Disease Questionnaire (CLDQ) is a commonly used HRQL tool in liver disease [7, 8]. Although NAFLD is mostly asymptomatic, patients may experience fatigue, emotional health impairment, and reduced activity, which may impact their HRQL [9]. There have been some studies focusing on HRQL for patients with NAFLD [9–11]. Sayiner et al. found that patients with NAFLD had significantly lower quality of life and health utility scores than the general population [9]. Golabi et al. used population-based data to assess HRQL in patients with NAFLD and also found that NAFLD was associated with impairment of HRQL [11]. Other study showed that weight loss can significantly improve the quality of life for patients with NAFLD [12]. Besides, quality of life is found to be a good indicator of improved functioning for people with chronic conditions [13]. However, national survey on HRQL of patients with NAFLD is seriously lacking in China. Therefore, the aim of this study was to assess the impact of NAFLD on HRQL in a Chinese population, and to investigate whether variables related to sociodemographic and clinical characteristics were independently associated with HRQL, and finally to identify independent risk factors of HRQL.

## Methods

### Study design and sample size

This was a national multicenter cross-sectional survey of HRQL in Chinese population with NAFLD. The sample was selected via convenience sampling method and patients with NAFLD in 90 hospitals among 24 provinces in China were recruited consecutively from March 1 to August 1, 2019.

### Participant

The diagnosis of NAFLD was based on the evidence of ultrasound, computed tomography (CT) and magnetic resonance imaging in 24 months or liver biopsies in 36 months. Patients at least 18 years of age were included in this study. Patients with other causes of liver injury (such as hepatitis B virus, hepatitis C virus or excess alcohol consumption [> 196 g/week for males, > 98 g/week for females], etc.), other causes of hepatic steatosis (such as tamoxifen or glucocorticoid), decompensated liver disease or hepatic cellular cancer, medical or psychological diseases, or impaired cognitive function were excluded in this study.

### Sociodemographic variables

Gender, age, residence, marital status, occupation, education (junior school, high school, college or above) and family income (less than local average, local average or more than local average) were self-reported. Laboratory examination included triglyceride (TG) and alanine transaminase (ALT). Body mass index (BMI) was calculated as weight in kilograms divided by the square of height in meters. Central obesity was defined as waist circumference ≥ 85 cm for female or ≥ 90 cm for male. Simple fatty liver, NASH and cirrhosis were reported or diagnosed by experienced hepatologists according to medical history and clinical examination of patients. In addition, complications (such as diabetes, hypertension, hyperlipidemia, cardiovascular disease, depression, and colon disease) were diagnosed by doctors.

### Assessment of HRQL

As a fully validated disease-specific instrument, CLDQ had been developed and widely used for patients with chronic liver disease [14]. The CLDQ-NAFLD instrument was used to assess HRQL of patients with NAFLD in this study. The CLDQ-NAFLD instrument had 36 items grouped into 6 domains: abdominal symptoms, activity, emotional, fatigue, systemic symptoms, and worry. All the questions were formulated as “How much of the time” or “How often you experienced a problem”, and a 1–7 Likert scale was introduced for the responses (the score of 1 would correspond to “All of the time”, and the score of 7 to “None of the time”). The overall CLDQ score was calculated as an average of all domains. Higher score on the questionnaire was indicative of minimum symptoms and lower score indicated more pronounced symptoms [7].

### Ethics

This study was approved by the Institutional Review Board of Peking University People’s Hospital (2018PHB259-01). Written informed consent was obtained from each patient.

### Statistical analysis

All statistical analyses were performed by using SPSS version 23.0 (SPSS Institute. IL.USA). Continuous variables were expressed as means ± standard deviations (SD), while categorical variables were expressed as number and percentage. Student t-tests (gender, central obesity and complications) and analysis of variance (age, residence, education, family income, BMI and disease severity) were used to compare the CLDQ score between groups. A stepwise multivariate logistic regression analysis was then used to identify the independent predictors that associate with HRQL. Statistical significance was set at *p* < 0.05.

## Results

### Sociodemographic and clinical characteristics

A total of 5181 patients with NAFLD were enrolled in this national multicenter cross-sectional survey. The sociodemographic and clinical characteristics of included patients were listed in Tables 1 and 2. Patients had a mean age of 43.8 ± 13.3 years and a mean BMI of 27.7 ± 6.0 kg/m^2^. The majority of patients in this study were male (65.8%), central obesity (71.8%), simple fatty liver (90.0%), and had a family income reached to local average (70.5%). The mean duration of ongoing treatment was 12.9 ± 25.9 months. Among the 5181 patients, 801 (15.5%) had diabetes, 1191 (23.0%) had hypertension, 1955 (37.7%) had hyperlipidemia, 414 (8.0%) had cardiovascular disease, 135 (2.6%) had depression, and 115 (2.2%) had colon disease.

**Table 1:** Sociodemographic characteristics of included patients

Sociodemographic characteristics	Patients (n = 5181)
Age (years), Mean ± SD	43.8 ± 13.3
Age group (n, %)	
18–39 years	2210 (42.6)
40–59 years	2256 (43.5)
≥ 60 years	715 (13.8)
Gender (n, %), male/female	3411 (65.8)/1770 (34.2)
Residence (n, %)	
East	1709 (33.0)
West	629 (12.1)
South	1539 (29.7)
North	1184 (22.9)
Central	119 (2.3)
Marital status (n, %)	
Single	647 (12.5)
Married	4407 (85.1)
Separated/divorced/widowed	127 (2.5)
Occupation (n, %)	
White collar	2505 (48.3)
Blue collar	746 (14.4)
Unemployed	720 (13.9)
Others	1210 (23.4)
Education (n, %), junior/high/college	1195 (23.1)/1266 (24.4)/2720 (52.5)
Family income (n, %)	
< Local average	339 (6.5)
Local average	3651 (70.5)
> Local average	946 (18.3)
Unknown	245 (4.7)

**Table 2:** Clinical characteristics of included patients

Clinical characteristics	Patients (n = 5181)
BMI (kg/m^2^), Mean ± SD	27.7 ± 6.0
BMI group (n, %)	
< 24 kg/m^2^	978 (18.9)
24–28 kg/m^2^	2363 (45.6)
≥ 28 kg/m^2^	1840 (35.5)
Central Obesity (n, %)	3720 (71.8)
Duration of NAFLD (mo), Mean ± SD	36.8 ± 46.5
Disease severity (n, %)	
Simple fatty liver	4661 (90.0)
NASH	187 (3.6)
Cirrhosis	28 (0.5)
Unknown	305 (5.9)
Complications (n, %)	
Diabetes	801 (15.5)
Hypertension	1191 (23.0)
Hyperlipidemia	1955 (37.7)
Cardiovascular disease	414 (8.0)
Depression	135 (2.6)
Colon disease	115 (2.2)
Duration of ongoing treatment (mo), Mean ± SD	12.9 ± 25.9
Laboratory examination, Mean ± SD	
ALT (U/L)	53.2 ± 47.0
TG (mmol/L)	2.4 ± 2.1

NAFLD, non-alcoholic fatty liver disease; NASH, non-alcoholic steatohepatitis; BMI, body mass index; SD, standard deviations; mo, months; ALT, alanine transaminase; TG, triglyceride

### CLDQ

Table 3 showed the CLDQ score in overall and six domains. The overall CLDQ score was 5.66 ± 0.89. Patients with NAFLD had impaired HRQL in all the six domains of CLDQ (abdominal symptoms, 5.59 ± 1.07; activity, 5.73 ± 0.95; emotional function, 5.66 ± 0.98; fatigue, 5.31 ± 1.10; systemic symptoms, 5.73 ± 0.92; worry, 5.88 ± 1.00).

**Table 3:** Chronic Liver Disease Questionnaire score in overall and six domains

Variable	Score (Mean ± SD)
Overall CLDQ score	5.66 ± 0.89
CLDQ domains	
Abdominal symptoms	5.59 ± 1.07
Activity	5.73 ± 0.95
Emotional function	5.66 ± 0.98
Fatigue	5.31 ± 1.10
Systemic symptoms	5.73 ± 0.92
Worry	5.88 ± 1.00

CLDQ, Chronic Liver Disease Questionnaire; SD, standard deviations

### Logistic regression analysis

**Fig. 1:**
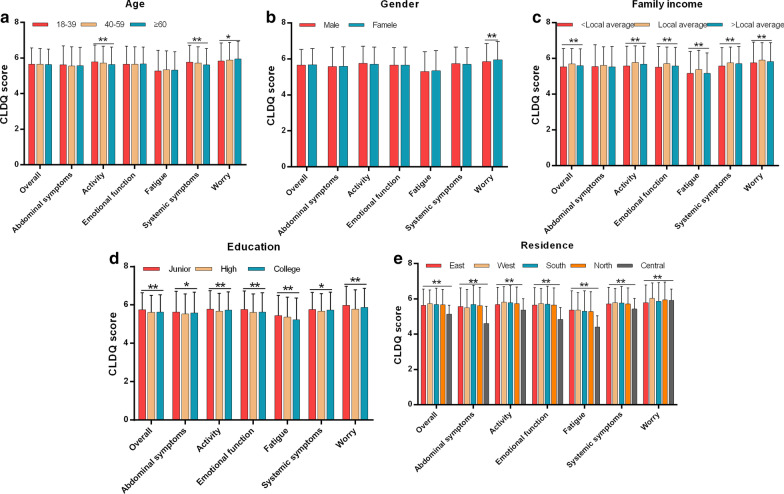
Univariate analysis of sociodemographic characteristics variables for the CLDQ score in overall and six domains. CLDQ, Chronic Liver Disease Questionnaire; *p < 0.05; **p < 0.01

**Fig. 2:**
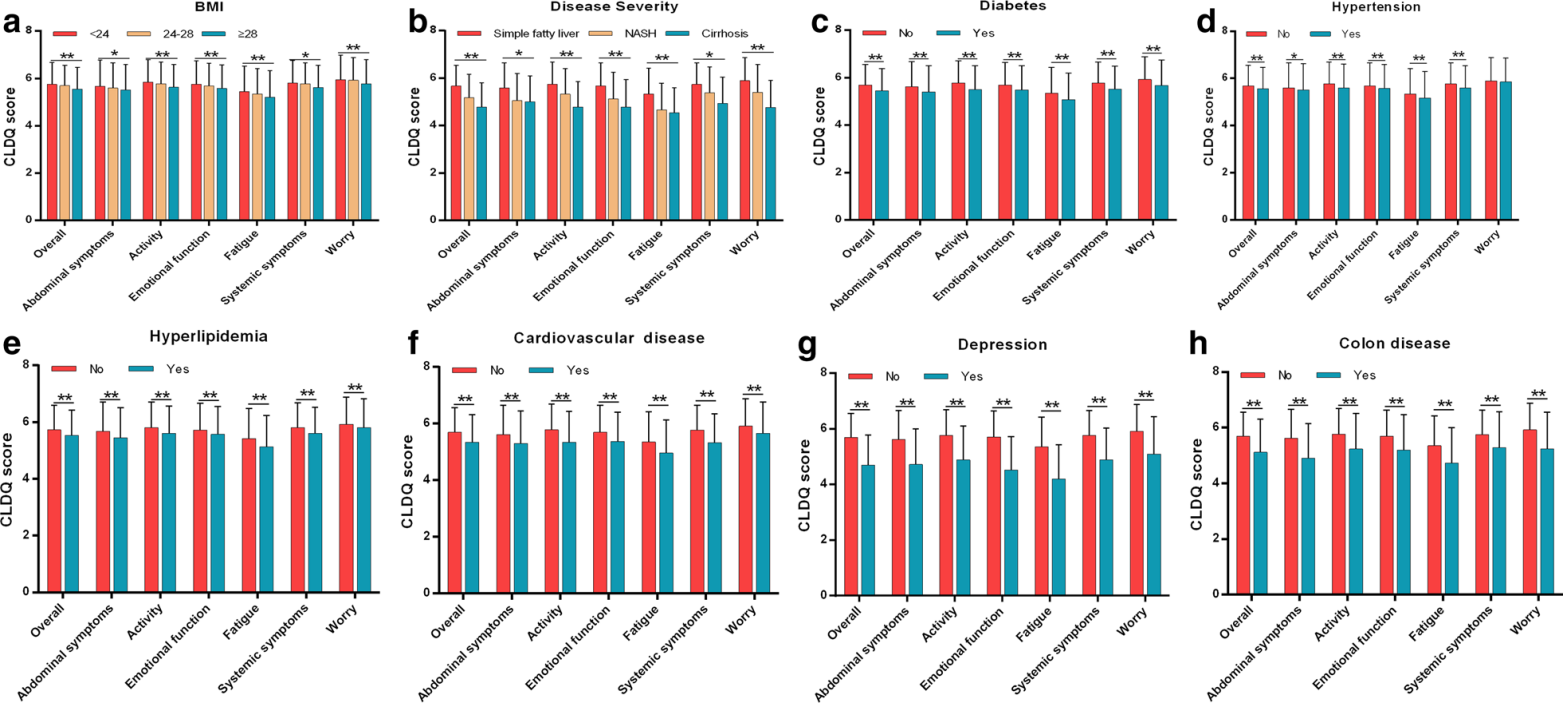
Univariate analysis of clinical characteristics variables for the CLDQ score in overall and six domains. CLDQ, Chronic Liver Disease Questionnaire; BMI, body mass index; *p < 0.05; **p < 0.01

**Fig. 3:**
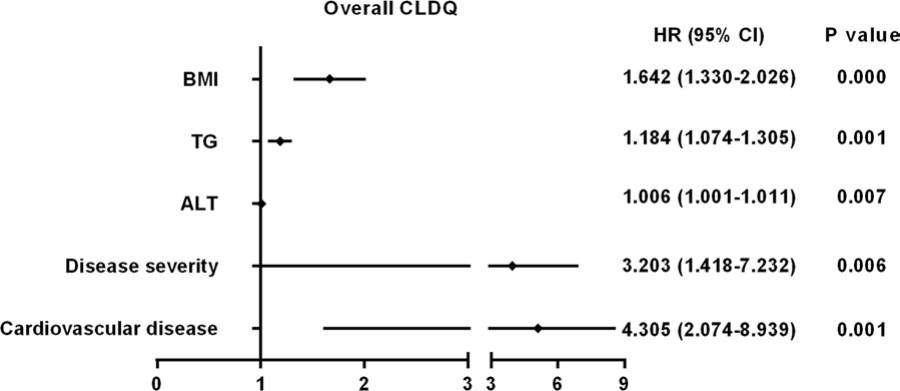
Multivariate logistic regression analysis of independent risk factors for overall CLDQ score. CLDQ, Chronic Liver Disease Questionnaire; BMI, body mass index; ALT, alanine transaminase; TG, triglyceride; 95% CI, 95% confidence interval; HR, hazard ratios

Univariate analysis revealed that family income, education, residence, ALT, TG, BMI, disease severity, and clinical complications including diabetes, hypertension, hyperlipidemia, cardiovascular disease, depression, colon diseases (all *p* < 0.05) were significantly associated with overall CLDQ score (Figs. 1 and 2). Multivariate logistic regression analysis showed that BMI (HR, 1.642; 95% CI, 1.330–2.026), ALT (HR, 1.006; 95% CI, 1.001–1.011), TG (HR, 1.184; 95% CI, 1.074–1.305), disease severity (HR, 3.203; 95% CI, 1.418–7.232), cardiovascular disease (HR, 4.305; 95% CI, 2.074–8.939) were independent risk factors for overall CLDQ score (Fig. 3).

**Fig. 4:**
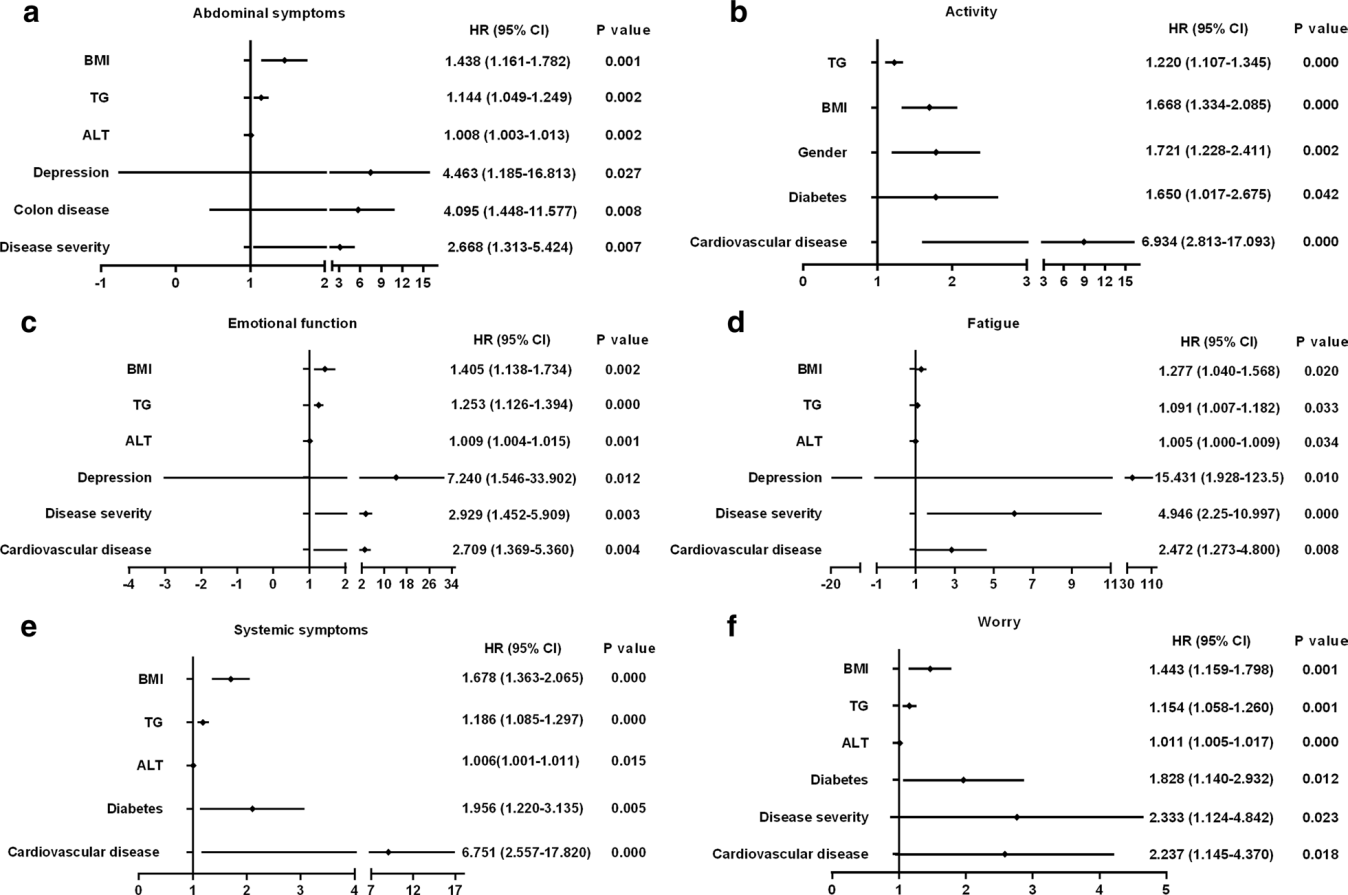
Multivariate logistic regression analysis of independent risk factors for the CLDQ score in six domains. CLDQ, Chronic Liver Disease Questionnaire; BMI, body mass index; ALT, alanine transaminase; TG, triglyceride; 95% CI, 95% confidence interval; HR, hazard ratios

In addition, univariate analysis revealed that education, residence, ALT, TG, BMI, disease severity, and clinical complications including diabetes, hyperlipidemia, cardiovascular disease, depression, colon disease were significantly associated with the CLDQ score of all domains (Figs. 1 and 2). Multivariate logistic regression analysis showed that BMI and TG were independent risk factors for the CLDQ score of all domains (Fig. 4). ALT, disease severity, diabetes, depression and cardiovascular disease were independent risk factors for the CLDQ score of several domains. Colon disease (HR, 4.095; 95% CI, 1.448–11.577) was the independent risk factor only for the CLDQ score of abdominal symptoms domain (Fig. 3a), while gender (HR, 1.721; 95% CI, 1.228–2.441) was the independent risk factor only for activity domain (Fig. 3b).

## Discussion

Since the number of patients with NAFLD in China may account for half of the patients with NAFLD worldwide, it is necessary to explore not only clinical outcomes but also quality outcomes, especially patient-reported outcomes (PRO) [5]. Although many different PROs are analyzed, HRQL is one of the most important [15]. Exploring the impact of NAFLD on patients’ well-being and HRQL is helpful to improve their care management. In this national multicenter cross-sectional survey in China, poor HRQL were demonstrated in these patients with NAFLD. BMI, ALT, TG, disease severity and cardiovascular disease were independent risk factors for HRQL. The present study from China may provide important evidence for expanding the knowledge about the effects of NAFLD on patients.

The study performed by Golabi et al. showed that 22% of patients with NAFLD reported that their health was poor or fair, which was significantly higher than that of the healthy control group (10%) [11]. Data from the Nonalcoholic Steatohepatitis Clinical Research Network (NASH CRN) showed that subjects with NAFLD had worse physical and mental health scores compared to the U.S. general population (with and without chronic illness) [16]. Similarly, Dan et al. demonstrated that patients with NAFLD had significantly lower HRQL compared with patients with hepatitis B or hepatitis C [17]. In present study, we also found that the HRQL in Chinese patients with NAFLD was impaired on all CLDQ domains, with an overall CLDQ score of 5.66 ± 0.89, even although majority of patients with NAFLD included in our study were asymptomatic.

The present study showed that disease severity was significantly correlated with the CLDQ score in overall and six domains, and the CLDQ score (simple fatty liver, 5.67 ± 0.87; NASH, 5.17 ± 1.00; cirrhosis, 5.17 ± 1.02) decreased with the progression of NAFLD. Although our study may underestimate the severity of NAFLD due to the self-reported diagnosis and the limited application of liver biopsies during clinical practice of NAFLD in China, the same trend existed in all six domains. David et al. reported that patients who had progressed to NASH had lower HRQL scores than patients with simple steatosis, and patients with cirrhotic had the least HRQL scores [16]. Another study showed that patients with cirrhosis had lower quality of life and utility scores than non-cirrhotic patients, however mental health scores did not differ between participants with and without NASH [18]. The divergence between our and previous study may be result from the socioeconomic environment of different countries, different HRQL tools and inclusion criteria.

In present study, we also found that the HRQL of patients with NAFLD worsened with the increase of BMI, and BMI was independent risk factor of the CLDQ score in overall and six domains. This was consistent with previous study, which showed that obese children with NAFLD had worse total, physical and psychosocial health scores compared to healthy children [19]. Basing on the significant association of BMI and HRQL of patients with NAFLD, some studies had tried to confirm the fact that the decrease of BMI could improve the impairment of HRQL [12]. Abdelbasset et al. indicated that an eight-week high-intensity interval aerobic exercise had a beneficial effect on weight loss and HRQL [20]. Hickman et al. also demonstrated that the HRQL of patients with NAFLD and HCV was significantly improved after weight loss [21]. Although patients with normal weight (BMI < 24 kg/m^2^), named as lean NAFLD, had highest CLDQ score than those of overweight (BMI 24–27.99 kg/m^2^) and obese patients (BMI ≥ 28 kg/m^2^), lean patients with NAFLD still had impaired HRQL according to our results. As previous studies had never reported the HRQL of lean patients with NAFLD yet, our results may provide some knowledge about the impairment of HRQL in these patients.

The key strength of the study is that this is a national multicenter HRQL survey of patients with NAFLD with a large sample size, which helps to obtain PRO information of patients with NAFLD in China. However, there are several limitations in the current study. Firstly, due to the large population of patients with NAFLD in China and most of them with no symptom, some patients may not be diagnosed, which makes it impossible to obtain the specific number of patients with NAFLD in China. Considering the feasibility of the study, the sample was selected via convenience sampling method and patients with NAFLD in 90 hospitals among 24 provinces in China were recruited consecutively from March 1 to August 1, 2019. Secondly, the CLDQ-NAFLD used in this study has not been well verified in China. However, CLDQ-NAFLD is still a better choice for us because it’s more specific for patients with NAFLD compared with CLDQ or Short Form 36-item Health Survey (SF-36). Thirdly, the validation population of CLDQ-NAFLD does not include patients with end-stage liver disease. In our study, only 28 patients with cirrhosis are included, but the number is too small to change the final results. Fourthly, the large sample size limits the performance of longitudinal follow-up. The lifestyle intervention, medicine and even surgery on improvement of HRQL of patients with NAFLD should form an important component of related studies in the future.

## Conclusions

This national multicenter cross-sectional survey in China indicated that the HRQL in patients with NAFLD was impaired. Significant association between HRQL and sociodemographic and clinical factors were found in this study. Attention should be paid to the optimally managing care of patients with NAFLD to improve their HRQL. The present study from China may provide important evidence for expanding the knowledge about the effects of NAFLD on patients. Since this is a cross-sectional survey, a longitudinal study on the impact of disease progression and interventions on HRQL is need in the future.

## Data Availability

All data generated or analysed during this study are included in this published article.

## Abbreviations

NAFLD: Non-alcoholic fatty liver disease
NASH: Non-alcoholic steatohepatitis
HRQL: Health Related Quality of Life
CLDQ: Chronic Liver Disease Questionnaire
CT: Computed tomography
TG: Triglyceride
ALT: Alanine transaminase
BMI: Body mass index
SD: Standard deviations
PRO: Patient-reported outcomes
